# Fish Allergy: Fishing for Novel Diagnostic and Therapeutic Options

**DOI:** 10.1007/s12016-020-08806-5

**Published:** 2020-07-25

**Authors:** D. Dijkema, J. A. M. Emons, A. A. J. M. Van de Ven, J. N. G. Oude Elberink

**Affiliations:** 1 Department of Nutrition and Dietetics, University Medical Center Groningen, House code AA34, PO Box 30.001, 9700 RB Groningen, The Netherlands; 2 Department of Pediatric Pulmonology and Allergology, Sophia Children’s Hospital/Erasmus Medical Center, Rotterdam, The Netherlands; 3 Department of Internal Medicine and Allergology, University Medical Center Groningen, Groningen, The Netherlands

**Keywords:** Fish allergy, Fish allergens, Parvalbumin, Enolase, Aldolase, Variable allergenicity

## Abstract

Fish allergy is one of the most common food allergies. The currently recommended treatment commonly consists of avoiding all fish species. Recent literature suggests that these recommendations are overprotective for the majority of fish-allergic patients. This review summarizes recent findings and provides practical information regarding management of fish allergy in the individual patient. After precise history taking supported by additional specific IgE measurements and/or skin prick tests, fish-allergic patients can generally be categorized into the following clinical clusters: (A) poly-sensitized patients reacting to all fish species due to their sensitization to the panallergen β-parvalbumin, (B) mono-sensitized patients with selective reactions to individual fish species only, and (C) oligo-sensitized patients reacting to several specific fish. A number of allergens including parvalbumin, enolase, and aldolase can be involved. Depending on the specific cluster the patient belongs to, oral food challenges for one or more fish species can be performed with the aim to provide safe alternatives for consumption. This way, several alternative fish species can be identified for mono- and oligo-sensitized patients that can safely be consumed. Notably, even poly-sensitized patients generally tolerate fish species low in β-parvalbumin such as tuna and mackerel, particularly when processed. Taken together, allergological evaluation of patients with a documented fish allergy should be strongly considered, as it will allow the majority of patients to safely reintroduce one or more fish species.

## Introduction

Fish is a valuable source of healthy nutrients such as omega-3 fatty acids and fat-soluble vitamins, and its consumption is increasing [1]. It is estimated that 0.1–0.4% of the world’s population has an allergy to fish [2, 3]. For this reason, fish has been included in the European mandatory labeling legislation together with 13 other allergens [4]. Patients with an allergy to one or more fish are in numerous allergy centers advised to avoid most or all fish species, a recommendation that in many cases turns out to be too strict. This article describes the current state of thoughts and guidelines with regard to diagnosis and treatment. It illustrates that patients with fish allergy can frequently still eat certain fish species.

There is a large biodiversity of fish, and there are considerable differences in fish consumption worldwide. While cod and salmon are widely consumed in Europe, freshwater fish are popular in Asia. To date, allergens from around 40 species of fish have been described. Sensitization can be caused by fish consumption but also by skin contact or by inhalation of fish steam during processing of fish [4]. The symptoms can vary from oral allergy syndrome, cutaneous involvement including angioedema, gastrointestinal symptoms such as nausea and vomiting, or anaphylaxis with respiratory and/or circulatory involvement [5–7].

An allergy to fish fillet is most prevalent, but allergic reactions have also been reported to fish roe (caviar), fish gelatin, and fish blood [4]. The allergens of fish fillet and fish roe differ so that patients who have an allergic reaction to fish roe can often eat fish fillet and vice versa [8]. To date, the allergenicity of fish gelatin, which is made from fish bone and/or fish skin, remains unclear [9, 10]. In only 3% of people with a known fish allergy or sensitization to tuna, IgE was found for fish gelatin made from tuna skin [9]. Out of 30 patients with an allergic reaction and demonstrable IgE sensitization to cod, only three patients had a positive skin test for fish gelatin made from cod. None of the 30 patients had an allergic reaction to fish gelatin made from cod skin during a food challenge test [10]. However, one case of anaphylaxis after ingestion of fish gelatin has been described in literature [11]. Fish blood seems to be a relevant allergen particularly for employees of fish processing companies, as its inhalation may cause occupational asthma.

## Fish Allergens

### Major Allergen: Parvalbumin

Most people who are allergic to fish have allergic reactions to multiple fish species [5]. This is explained by the high cross-reactivity of β-parvalbumin, the most important fish allergen present in various fish species (Table 1) [16–18]. The amino acid sequence of the different components of fish parvalbumin varies substantially (55–95%) between the different fish, but nevertheless, structural similarity remains [19]. β-Parvalbumin is an extremely thermostable allergen with a low molecular weight (10–12 kDa). In addition to the β-parvalbumin variant, cartilaginous fish (sharks and rays) contain the α-parvalbumin variant [20]. Despite the great homology between fish α- and β-parvalbumin, the allergenicity of fish α-parvalbumin is generally considered to be very low and well-tolerated,[21] possibly due to its close resemblance to human α-parvalbumin [19, 22]. Nevertheless, there are some case reports describing an allergic reaction to α-parvalbumin [19, 23].

**Table 1:**
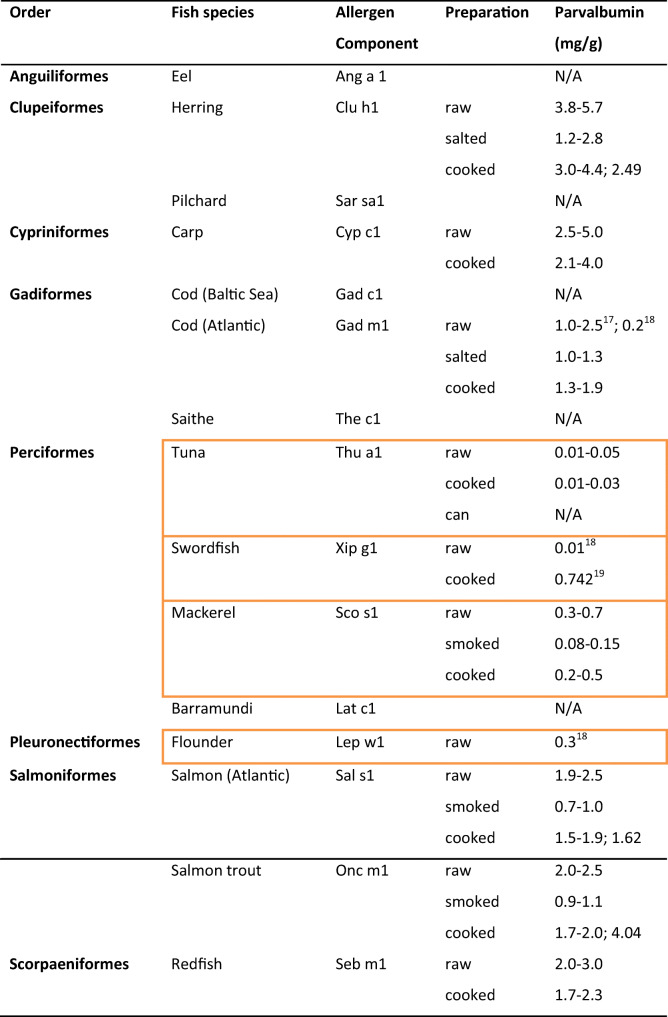
Overview of parvalbumin allergy [12–15]

The orange marking indicates the fish species with the lowest amount of parvalbumin

*N/A* not available

### Parvalbumin as a Panallergen

Despite the high structural resemblance of β-parvalbumin, only 59% of people with allergies to β-parvalbumin have a response to various fish species. This indicates that 41% of patients tolerate one or more fish species [24]. Despite certain common beliefs, tolerance of fish species is not related to the color of the fish’s flesh but is explained by the amount of β-parvalbumin in the muscles. Fish muscles consist of sarcomeres with light (actin, I-band, fast movements) and dark bands (myosin, A-band, continuous movements). Active fish, such as tuna and swordfish, have more dark bands in their muscles than demersal fish such as cod, flounder, and flatfish [14]. The amount of parvalbumin in the light bands is 4–8 times greater than in the dark bands [25]. Furthermore, it appears that the dorsal muscles with light bands contain more parvalbumin than the ventral muscles with light bands and the rostralated muscles with light bands contain more parvalbumin than the caudal muscles with light bands [15].

As a result, there is a difference in the amount of parvalbumin in the different fish species. Kuehn et al. found on average < 0.05 mg/g β-parvalbumin in tuna; 0.3–0.7 mg/g in mackerel; 1–2.5 mg/g in salmon, trout, and cod; and > 2.5 mg/g in the case of carp, herring, and redfish (Table 1) [13]. Apart from these intrinsic differences in parvalbumin quantity between fish species, the amount of parvalbumin also influenced the preparation method. Parvalbumin decreases due to the processing of fish through cooking, brining, and/or smoking. Due to these variations, it is possible that people with a clinically relevant sensitization to parvalbumin can still eat processed fish with a lower parvalbumin concentration.

Of note, it appears that IgE sensitization for different fish species based on parvalbumin does not correlate well with the reported clinical allergy or tolerance of the patient. Serological cross-reactivity with other fish species is therefore not always associated with clinical cross-reactivity [24].

### Parvalbumin as a Fish-Specific Allergen

Despite case reports of mono-sensitization for single fish species, until recently, there was no allergen-based explanation for these observations. For many years, sensitization for β-parvalbumin was linked to a cross-reactivity for several fish species. However, some parvalbumin species-specific epitopes have now been identified [26]. Patients with a clinically relevant mono-sensitization for Salmonidae recognize of the entire β-parvalbumin repertoire only the specific epitope (beta-1) of salmon parvalbumin (see Fig. 1 in Ref 27) [26–28]. Nine out of 62 fish-allergic patients (15%) only experience allergic reactions to salmonids [29]. A parvalbumin epitope has also been discovered for pangasius, catfish, and monkfish, which explains the same clinical mono-sensitivity [30]. This suggests that parvalbumin-based cross-reactivity can be limited to one or a few closely related species of fish.

**Fig. 1:**
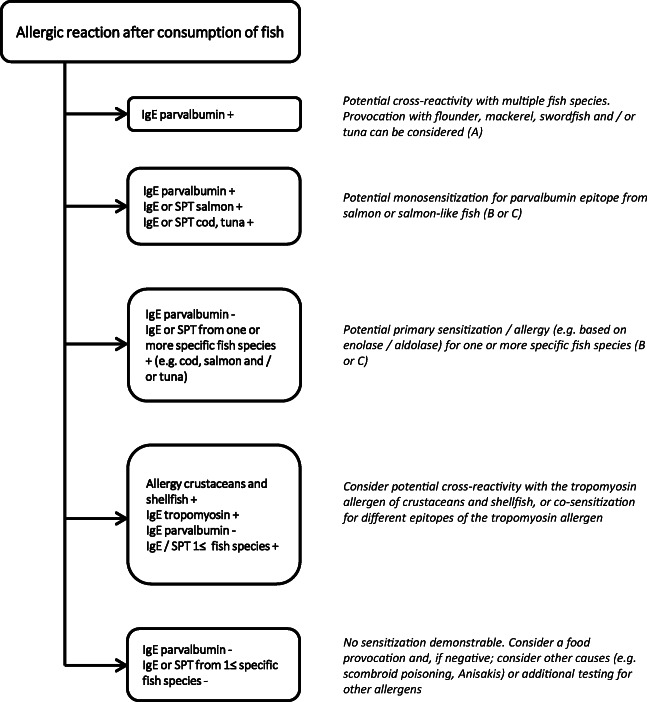
Flow chart of recommended diagnostics following an allergic reaction after fish consumption. SPT, skin prick test

### Other Fish Allergens: Enolase and Aldolase

In 2013, the 47–50 kDa enolase and 40 kDa aldolase protein families were identified as major heat-labile fish allergens in cod, salmon, and tuna (Table 2).

**Table 2:** Overview of enolase, aldolase, tropomyosin, and vitellogenin allergens [12]

Order	Fish	Allergen component	Protein family
Gadiformes	Cod (Atlantic Ocean)	Gad m2	Enolase
		Gad m3	Aldolase
Perciformes	Tuna	Thu a2	Enolase
		Thu a3	Aldolase
	Tilapia	Ore m4	Tropomyosin
	Salmon	Onc k5	Vitellogenin
Salmoniformes	Salmon (Atlantic Ocean)	Sal s2	Enolase
		Sal s3	Aldolase

The enolase and aldolase protein families were initially associated with a specific fish allergy, but a high number of fish-allergic patients with IgE for enolase and aldolase were also found to have IgE for parvalbumin [29]. However, there are also some case reports describing patients who lack parvalbumin-specific IgE but do have a clinically relevant mono- or oligo-sensitization for specific fish [4, 31]. A clinically relevant sensitization for enolase or aldolase, in the absence of parvalbumin-specific IgE, therefore appears to be associated with a species-specific fish allergy [32]; nevertheless, cross-reactivity between enolase and aldolase allergens cod, salmon, and tuna remains possible [29]. Until now, the relevance of enolase and aldolase as thermolabile food allergens has not been sufficiently specified, particularly for parvalbumin-negative patients [8].

### Other Fish Allergens: Vitellogenin and Tropomyosin

In addition to the abovementioned allergens, two other fish allergens are registered in the IUIS database: vitellogenin and tropomyosin (Table 2). Vitellogenin, a protein in fish roe, is registered as a food allergen in caviar from various fish [33, 34]. Tropomyosin, known as a thermostable panallergen in crustaceans, was discovered as an allergen in 2013 in patients with an allergy to tilapia [35]. Although it is thought that tropomyosin from crustaceans and shellfish does not cross-react with that of fish, a tropomyosin allergen may seem to play a role in allergic reactions to cod, albacore tuna [36], swordfish, monkfish, flatfish, and hake [37]. This suggests possible cross-reactivity between crustaceans/shellfish and fish or co-sensitization for different epitopes of the tropomyosin allergen. In contrast with other fish, much less is known about allergens for tropical freshwater fish, for example, tilapia species (*Oreochromis* genus). Recently, other allergens such as parvalbumin, enolase, aldolase, and other unknown kDa proteins were found in tilapia [35, 38, 39]. Allergens different from parvalbumins appear to be more important for tropical freshwater fish [35, 39]. It is unclear whether tilapia cross-reacts with other fish species or shrimp. Based on similarity of the tilapia tropomyosin allergen Ore m4, which is 58.8% identical to the northern shrimp (*Pandalus borealis*) tropomyosin and 57.8% identical to the house dust mite (*Dermatophagoides farina*) tropomyosin, cross-reactivity could be expected.

In addition to vitellogenin and tropomyosin, some fish allergens with unclear clinical relevance have been reported [38, 40, 41]. Allergens have been found in swordfish: 25 kDa [31] and 28, 33, 38, 38, and 57 kDa [42]. Only the 28 kDa and 57 kDa allergens could be identified: the 28 kDa allergen as triose phosphate isomerase and the 57 kDa allergen as pyruvate kinase. In addition to these allergens, swordfish also contains a 50 kDa enolase, a 40 kDa aldolase allergen, and a 14 kDa parvalbumin allergen [42]. There are two case reports describing a patient with a specific allergy to swordfish and tolerating other fish species, but the relevant allergenic culprit remains unidentified [31, 42]. Importantly, food challenges can be positive also in the absence of swordfish-specific IgE ([42]; see clinical case 3), and the same holds true for pan herring allergy [43]. These findings suggest that the sensitivity of commercially available IgE blood tests of certain fish species is insufficient and/or lack certain unidentified clinically relevant minor allergens.

## Recommendations for Clinical Practice

When evaluating fish allergy, we find it helpful to categorize patients with fish allergy into three clusters: (A) poly-sensitized patients who respond to all types of fish on the basis of cross-reactions of β-parvalbumin and often enolase and aldolase, (B) mono-sensitized patients with a selective allergic reaction for one individual fish species based on a specific epitope of β-parvalbumin, and (C) oligo-sensitized patients who respond to a number of specific fish based on enolase and aldolase, without IgE for β-parvalbumin [29, 32, 44].

Other recommendations include:

1. Refer to an allergy dietitian for evaluation and counseling.
2. Establish a presumptive diagnosis based on history and additional allergy tests.
3. Discuss the patient’s perspective regarding further diagnostics (particularly related to 4) and wish for consumption.
4. Perform oral food challenge(s) after shared decision-making.

### Ad 1

The role of a specialized allergy dietitian is multifold, supporting diagnostic evaluation, assistance in oral food challenges, and counseling of an established fish allergy. We therefore strongly encourage early referral to the allergy dietitian, so she/he can take a precise and comprehensive food history focused on food allergy and assisting in the (diagnostic) avoidance of one, a few, or all fish species. Second, the allergy dietitian is pivotal in the development and implementation of the oral food challenges and can advise the allergist which protocols to use. At a later stage, i.e., in the event of a suspected or confirmed fish allergy, the dietitian offers guidance regarding the intake of important omega-3 fatty acids. Examples of alternative sources of omega-3 fatty acids—other than the fish species they do tolerate—are shellfish, rapeseed, linseed, soybean oil, nuts, and vegetable oil-based (low-fat) margarine. If necessary, supplementation of fish oil capsules can be considered, as well as more costly algae-based supplements. Moreover, the dietitian offers guidance in dealing with the risk of cross-contamination for the fish species the patient is allergic to, which is important for any patient with fish allergy, regardless of which type (cluster A, B, or C).

### Ad 2

The presumptive diagnosis of fish allergy can generally be made based on a precise history combined with additional specific IgE testing and/or skin tests. If this does not provide sufficient clarity, an open or preferably double-blinded food challenge test with the suspected culprit fish is advised (see Ad 4). To date, the repertoire of diagnostic tests for certain fish species that are commercially available is still limited, and the use of prick-to-prick tests with homemade fish extracts is often the only possibility. For optimal preparation of these extracts, collecting material from the dorsal-rostral part of the fish is recommended in case of suspicion of a parvalbumin allergy [21]. In line with other food allergies, diagnostic tests should always be interpreted with caution and always in combination with the clinical history. Especially for cluster A (poly-sensitized patients to β-parvalbumin), but also for the other cluster, serological cross-reactivity with other fish species is not necessarily clinically relevant [24]; conversely, negative blood tests cannot entirely rule out an allergy, as will be demonstrated in clinical case 3 for swordfish [42, 43]. Hence, in case of uncertainty, an oral food challenge is recommended.

### Ad 3

From the patient’s point of view, complete certainty regarding the culprit is not always essential, as they often have developed an aversion for fish and patients are fine by a diet free of fish. In our experience however, the majority prefers to know whether there are fish species that they might be able to consume safely, as they are aware that fish is valuable source of healthy nutrients or simply like the taste of fish. Therefore, it is important to be aware that most fish-allergic patient can still tolerate selected fish species, even in the case of poly-sensitization (cluster A). In case the medical history does not provide sufficient clarity, an IgE screening of parvalbumin (e.g., Gad c1) is recommended, followed by specific IgE and/or skin tests of both the fish species to which the patient reacted and the fish species that the patient would like to eat (Fig. 1). Based on current knowledge about the three general fish-allergic clusters (A, B, and C) and the quantity and type of protein in different fish species for cluster A, an individual patient specific advice can be given (Table 3).

**Table 3:** General recommendations for oral food challenge, which fish species can potentially be safely consumed if the patient is allergic for fish cluster A, B, or C

Fish-allergic cluster	Oral food challenge with:
A	Most likely: tuna. Other: mackerel, swordfish, or flounder
B	- If only allergic to the salmon-specific epitope β-parvalbumin: any fish species that is not of the Salmonidae family - If allergic to another specific β-parvalbumin epitope (pangasius, catfish and monkfish): any fish species that is not the culprit fish family
C	Sufficiently heated fish species

### Ad 4

The decision of which oral food challenge should be performed depends on the fish-allergic cluster (A, B, or C) the patient is in, which fish species contains most valuable source of healthy nutrients and which fish species is preferred by the patient (see Ad 3). Usually, one or a few oral food challenges would suffice to establish both a diagnosis and provide safe alternatives. Since it is not always clear which fish species is processed in food (i.e., some brands of fish broth), reading EU food labels remains necessary. Ideally, EU labeling with the specific fish species instead of general labeling would facilitate expanding the diet of a fish-allergic person and perhaps even reduce anxiety when consuming products outside their normal daily routine.

Since it remains challenging to provide general recommendations on the indication of an oral food challenge, we added three clinical cases that illustrate the relevance of performing additional tests to further specify the fish allergy.

### Case 1

A 22-year-old man experienced symptoms of oral pruritic and burning sensations, shortness of breath, and ocular itching twice within 6 months, once after eating salmon and once after eating cod. The reactions arose fairly quickly after taking a few bites of the fish species. Contact with salmon and cod on the hands also caused itching. Prior to the allergic reactions, he tolerated all types of fish. After the allergic reactions, he had avoided all fish except tuna, which he could eat without any symptoms. Diagnostic allergological evaluation showed a poly-sensitization pattern: IgE cod 27 kU/L, IgE cod Gad c1 51 kU/L, IgE tuna 7.2 kU/L, and IgE salmon 31 kU/L. The allergen parvalbumin (Gad c1) was positive, making an allergy to the majority of the fish species (cluster A) seem plausible given the cross-reactivity.

Since fish with a low concentration of parvalbumin (tuna, < 0.05 mg/g parvalbumin) were tolerated but a reaction occurred at higher concentrations (salmon, cod; see Table 1), an oral food challenge with another fish species with a low concentration of parvalbumin was proposed. The patient received a 6-step open challenge with smoked mackerel (0.08–0.15 mg/g parvalbumin). The challenge was completed uneventfully; the patient ate the total amount of 80 g of mackerel without experiencing symptoms. He was advised to avoid all fish species except tuna and mackerel. Additional challenges with other fish species such as swordfish and flounder can be considered.

### Case 2

A 16-year-old atopic girl experienced buccal blistering and pain with angioedema shortly after eating salmon. She could eat fish fingers (coal fish), squabbling, tuna, and pangasius fillet without any problems but had never eaten any other fish. Additional laboratory tests showed specific IgE for salmon 9.89 kU/L, tuna < 0.10 kU/L, cod 0.46 kU/L, and parvalbumin 1.13 kU/L. Prick-to-prick skin tests using extracts of raw fish species showed a strong response to salmon (histamine equivalent prick (HEP) 4.41) and weaker or no responses to other fish species (mackerel HEP 0.7, cod HEP 0.55, pangasius fillet HEP 0.51, herring HEP 0.33, coalfish HEP 0, catfish HEP 0, sole HEP 0, sole HEP 0, tilapia fillet HEP 0, eel HEP 0). Considering these diagnostics, an allergy to the salmon-specific parvalbumin epitope (cluster B) seems most plausible. Open food challenges with cod and mackerel were carried out, both of which were negative. The sensitization to pangasius fillet was not clinically relevant as this fish species was tolerated. Thus, a specific allergy to salmon was diagnosed, and the patient was advised to only avoid salmon.

### Case 3

A 40-year-old woman was referred after having a systemic reaction following a meal consisting of swordfish, white wine, anchovy fillet, and capers. The reaction included facial erythema, hives, pruritus, edema of lips and glottis, nausea, chest tightness, and tachycardia. As she tolerated other fish species without symptoms, an initial diagnosis of idiopathic anaphylaxis was made. Two years later however, she presented with a clinically identical reaction following a meal of tzatziki, swordfish, green beans, potatoes, and salad. Between the two reactions, she had consumed salmon, cod, and tilapia fillet without any problems. An ISAC (Immuno Solid-phase Allergen Chip) screening was performed. At that time, only parvalbumin of cod (Gad c1) of the fish allergens was in the ISAC, and this was negative (< 0.3 ISU-E). Anisakis allergen Ani s1 was also negative (< 0.3 ISU-E). Specific IgE for swordfish remained negative (< 0.10 kU/L).

An open challenge with swordfish was performed; the patient developed a tingling sensation on her lips, followed by erythema of face and neck and urticaria on her arms. She had eaten 50.45 g of swordfish cumulatively during this challenge. The test was considered positive, and the patient was advised to solely avoid swordfish as in this case, an allergy to one single fish species (with sensibilization to tropomyosin or an unknown allergen) seems most plausible. An allergy to enolase and aldolase is less likely because of their heat instability; an allergy to parvalbumin is unlikely since swordfish is a fish species low in parvalbumin.

The last case illustrates the added value of an oral food challenge in case of a high clinical suspicion of fish allergy, even if specific IgE is lacking. The main differential diagnosis of fish allergy consists of scombroid poisoning, particularly if the fish species had been tolerated very recently, if the storage temperature of the fish had been inappropriate, and if multiple people who consumed the fish product experience similar symptoms. Allergic reactions to Anisakis simplex should also be considered, as this parasite can be present in raw or undercooked fish [8].

**Table:** 

Summary of instructions for practice	
1. Patients with a fish allergy can be divided into three clusters: (A) poly-sensitized patients who respond to all types of fish, (B) mono-sensitized patients with a selective allergic reaction to one individual fish species, and (C) oligo-sensitized patients who respond to a number of specific fish. 2. The majority of patients with a fish allergy can still eat certain fish species. Given the health benefits of fish, it is important to prevent a patient from unnecessarily avoiding fish. 3. If a parvalbumin-based allergy is suspected, for a skin test, it is advisable to collect material from the dorsal-rostral part of the fish. 4. Tropomyosin is known as the panallergen in crustaceans and shellfish. There are indications that the tropomyosin allergen also plays a role in some fish species, suggesting that there might be cross-reactivity between fish and crustaceans/shellfish.	

## Data Availability

Not applicable.
